# Strength–Ductility Mechanism of CoCrFeMnNi High-Entropy Alloys with Inverse Gradient-Grained Structures

**DOI:** 10.3390/ma17071695

**Published:** 2024-04-07

**Authors:** Jie Chen, Yongqiang Hu, Pengfei Wang, Jingge Li, Yu Zheng, Chengtong Lu, Bohong Zhang, Jiahai Shen, Yu Cao

**Affiliations:** 1 School of Information Science and Technology, Northwest University, Xi’an 710127, China; 2 Zhejiang Provincial Key Laboratory of Laser Processing Robotics, College of Mechanical & Electrical Engineering, Wenzhou University, Wenzhou 325035, China; 3 China International Science & Technology Cooperation Base for Laser Processing Robotics, Wenzhou University, Wenzhou 325035, China; 4 Zhejiang Wuma Reducer Co., Ltd., Wenzhou 325019, China; 5 Sichuan University-Pittsburgh Institute, Sichuan University, Chengdu 610207, China; 6 Haining Textile Machinery Co., Ltd., Haining 314400, China

**Keywords:** high-entropy alloy, laser surface heat treatment, inverse gradient-grained structure, strength–ductility synergy, hierarchical nanotwins

## Abstract

The microstructures and mechanical properties of equiatomic CoCrFeMnNi high-entropy alloys (HEAs) treated with various processing parameters of laser surface heat treatment are studied in this paper. The typical inverse gradient-grained structure, which is composed of a hard central layer and a soft surface layer, can be obtained by laser surface heat treatment. A much narrower gradient layer leads to the highest yield strength by sacrificing ductility when the surface temperature of the laser-irradiated region remains at ~850 °C, whereas the fully recrystallized microstructure, which exists from the top surface layer to the ~1.05 mm depth layer, increases the ductility but decreases the yield strength as the maximum heating temperature rises to ~1050 °C. Significantly, the superior strength–ductility combination can be acquired by controlling the surface temperature of a laser-irradiated surface at ~1000 °C with a scanning speed of ~4 mm/s due to the effect of hetero-deformation-induced strengthening and hardening, as well as the enhanced interaction between dislocation and nanotwins by the hierarchical nanotwins. Therefore, retaining the partial recrystallized microstructure with a relatively high microhardness in the central layer, promoting the generation of hierarchical nanotwins, and increasing the volume proportion of gradient layer can effectively facilitate the inverse gradient-grained CoCrFeMnNi HEAs to exhibit a desirable strength–ductility synergy.

## 1. Introduction

Gradient-grained structures, characterized by significantly disparate mechanical properties from the surface to the interior regions, are considered beneficial for overcoming strength–ductility trade-offs [1,2,3]. Generally, the gradient-grained structure can be divided into two kinds according to the distribution characterization of the grain size. One is the grain size that changes from nanoscale in the topmost surface to micron degree in the central layer by surface plastic deformation, such as surface mechanical attrition treatment [4]. Usually, such a grain size distribution is defined as a normal gradient-grained structure. However, the thickness of the layer with a gradient-grained structure is limited to hundreds of microns [5,6], which indicates that the surface mechanical treatment may not suitable for relatively thicker HEAs. In order to solve this problem, an inverse gradient-grained structure with a gradient-structured layer in millimeters is obtained by cold-rolling and subsequent electro-magnetic induction heating or laser surface heat treatment [5,7]. It is worth noting that the grain size in the inverse gradient-grained structure gradually decreases from a soft surface to a hard core, which is completely opposite to the normal gradient-grained structure. Moreover, the synergistic effect in inverse gradient-grained structures is not inferior to that of normal gradient-grained structures [5].

Although it is widely accepted that the strength–ductility synergy of gradient-grained materials is determined by their structural gradients, designing gradients remains a great challenge to achieve superior mechanical properties [4]. At present, most of the studies about the correlations between gradient-grained structures and strength–ductility combinations are focused on normal gradient-grained materials. Hasan et al. proposed that enlarging the strength difference between the surface and the undeformed core will significantly improve the strength–ductility synergy [8]. In their opinion, it is because the higher strength incompatibility in the adjacent layers can increase the magnitude of the strain gradient and the density of the geometrically necessary dislocation [9,10], which finally enhance the effects of hetero-deformation-induced strengthening and strain hardening, producing better mechanical properties [11,12]. However, Wang et al. found that the strain gradient-related strengthening effect does not increase linearly with an increasing strain gradient in the interface affected zone due to the dynamical formation and disappearance of geometrically necessary dislocation pileups [9]. Currently, owing to the lack of a quantitative relationship between the grain-size distribution characteristics, geometrically necessary dislocation density, hetero-deformation-induced strengthening, and strain hardening, there is no unified design principle for the normal gradient-grained materials to enhance their strength and ductility. For the inverse gradient-grained structure, the superior combination of strength and ductility is mainly attributed to the gradient-grained layer in the surface, along with the high-order hierarchical nanotwins in the hard core [7]. After reviewing a large number of domestic and foreign studies, we found that the effect of inverse gradient-grained structures on the strength–ductility synergy was rarely studied, and how to optimize the inverse gradient-grained structure remains a mystery.

In the present work, the cold rolling + laser surface heat treatment technique is utilized to create various grain size distributions by adjusting the laser power and scanning speed. Considering that grain coarsening occurs mainly at the surface layer of the ~3.5 mm thick cold-rolled CoCrFeMnNi HEAs under laser-beam irradiation in our previous study, accompanied by the nano- or submicron-scaled grains in the central region [7], the same HEAs are chosen as the experimental material. According to the comparison between three gradient-grained CoCrFeMnNi HEAs in terms of microstructural characteristics and strength–ductility synergy, the effect of the grain-size gradient on the mechanical properties is intensively investigated, and finally, the design principles of the inverse gradient-grained structure for improving the combination of strength and ductility are proposed.

## 2. Materials and Methods

The HEAs with a nominal composition of Co_20_Cr_20_Fe_20_Mn_20_Ni_20_ (in atomic percent, at.%) were prepared by arc melting using a mixture of pure metals (purity ≥ 99.7 wt.%). The as-cast CoCrFeMnNi HEAs were remelted at least three times to ensure chemical homogeneity. Subsequently, the cylindrical ingots with a diameter of ~96 mm were hot forged into slabs with a thickness of ~30 mm at ~1100 °C. After annealing at ~1100 °C for ~1 h, the slabs were further cold-rolled from a thickness of ~30 mm to ~3.5 mm thick sheets on the two-high reversing rolling mill.

The semiconductor laser heat treatment device was applied to process the inverse gradient-grained structure for the experimental CoCrFeMnNi HEAs. In order to precisely control the surface temperature of the laser-irradiated region, the two-color pyrometer together with the closed-loop control system can adjust the laser power in real time to acquire a stable and uniform heating temperature. Meanwhile, the maximum output power and spot size of this semiconductor laser heat treatment unit are ~4 kW and ~8 mm × ~6 mm, respectively. During laser surface heat treatment, the flat-topped beam scanned the upper and lower surfaces of the cold-rolled CoCrFeMnNi HEA sheets along the rolling direction (the ~6 mm direction of the spot was parallel to the rolling direction), and the surface temperature of the laser-irradiated region and the scanning speed were changed to obtain various inverse gradient-grained structures. In particular, three groups of technological parameters of laser surface heat treatment were observed in this study, such as 850-5 (inverse gradient-grained structure surface temperature of laser-irradiated region-scanning speed, similarly hereinafter), 1000-4, and 1050-5. In addition, finite element simulation was employed to characterize the temperature distribution along the depth of the experimental samples during laser heat treatment by using the ANSYS APDL 19.2 software.

Specimens for electron backscattering diffraction (EBSD, Oxford Symmetry S3, Oxfordshire, UK) observation were electropolished by electrolyte consisting of perchloric acid and ethyl alcohol (1:7) with a potential of ~25 V for ~20 s. The scanning step of the EBSD map was ~0.02 μm. AztecCrystal was applied to eliminate the point of zero resolutions and process the EBSD data. Transmission electron microscopy (TEM) tests were performed by FEI Tecnai G^2^ F20 (Hillsboro, OR, USA), and samples for TEM observation were ~3 mm diameter foils and were prepared by the twin-jet electropolished method with electrolytes composed of ~10% perchloric acid and ~90% alcohol.

The microhardness variation map of the gradient samples along the depth direction from the laser scanning surface were obtained by a Vickers microhardness tester (Mitutoyo HM-210B, Kawasaki, Japan) under a load of ~0.5 kgf and a duration of ~15 s. The microhardness values for the same layer were measured 10 times. Uniaxial tensile tests at room temperature were tested by the Instron 3369 testing machine equipped with a non-contacting video extensometer (Norwood, MA, USA) at a constant strain of ~5 × 10^−3^ s^−1^. Loading–unloading–reloading tests at room temperature were conducted to characterize the hetero-deformation-induced (HDI) stress during deformation, and the strain rate was set up as ~5 × 10^−4^ s^−1^.

## 3. Results and Discussion

### 3.1. Distribution Characteristics of Grain Size

**Figure 1:**
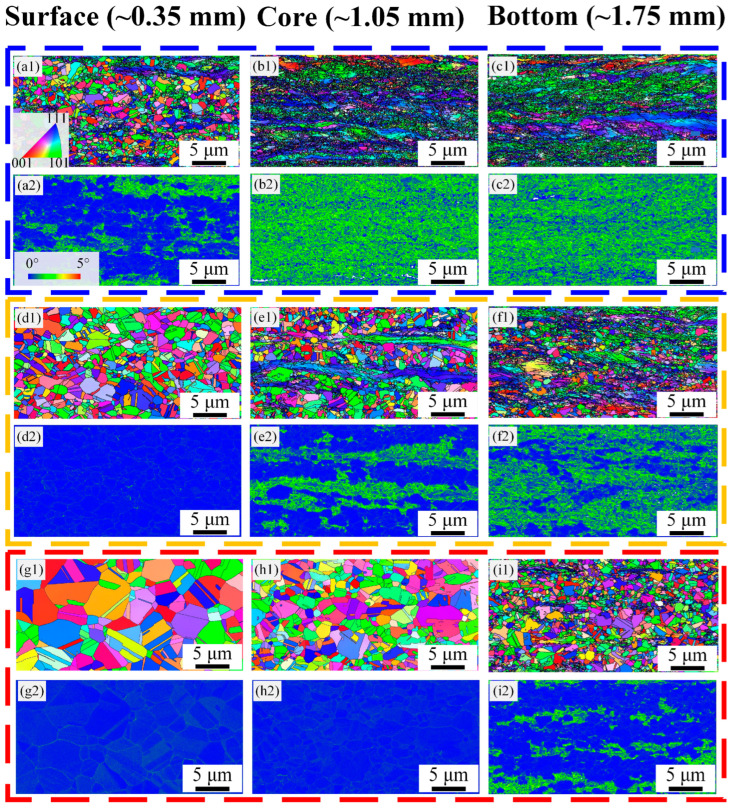
The IPF and KAM maps of the experimental CoCrFeMnNi HEAs at different depths: (**a**–**c**) 850-5 sample, (**d**–**f**) 1000-4 sample, and (**g**–**i**) 1050-5 sample.

Inverse gradient-grained structures of the 850-5, 1000-4, and 1050-5 samples, as depicted in Figure 1, are obtained, ascribed to the various degrees of recrystallization and growth of the grains at different depths induced by the laser surface heat treatment. Specific microstructural characteristics of the gradient-distributed grain and dislocation are shown in the inverse pole figure (IPF) maps, kernel average misorientation (KAM) map, and corresponding misorientation angle maps of the grain boundaries. The average KAM values are calculated by discarding the points with KAM values larger than ~2° [13].

**Figure 2:**
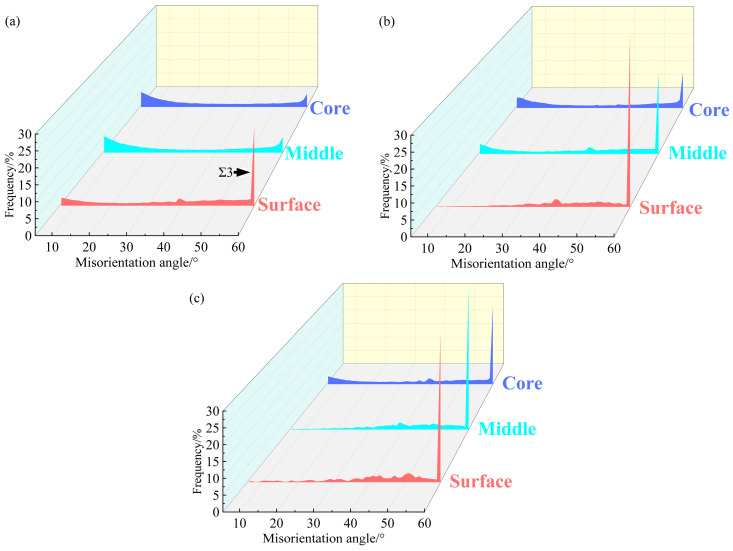
The distribution of misorientation angle of the inverse gradient-grained CoCrFeMnNi HEAs: (**a**) 850-5 sample; (**b**) 1000-4 sample; (**c**) 1050-5 sample.

The EBSD observation of the 850-5 sample is demonstrated in Figure 1a–c. The surface layer of the 850-5 sample is mainly composed of recrystallized grains at a micron scale, accompanying the fraction of annealing twin boundaries (*fΣ3)* of ~24.7% (Figure 2a). Meanwhile, the ultra-fine grains and lamellae with relatively high KAM values can also be observed in Figure 1(a2), which indicates that partial recrystallization occurs at the surface layer of the 850-5 sample. By increasing the depths, hardly any recrystallized grains can be seen in the ~1.05 mm depth layer (Figure 1b), as well as the central layer (Figure 1c), and both these layers are made up of deformed microstructures induced by the previous cold rolling. Therefore, *fΣ3* exhibits an extremely low level in the ~1.05 mm depth layer and the central layers of the 850-5 sample with the values of ~5.3% and ~4.7%, respectively. It can be easily deduced that the thickness of the gradient layer with the characteristics of gradient variation for the 850-5 sample is relatively thin due to the large amount of non-recrystallized grains in the ~1.05 mm depth layer and central layer. For the 1000-4 sample, a wider gradient layer is obtained compared to the 850-5 sample. As shown in Figure 1(e2), a fraction of the fully recrystallized grains for the surface layer reaches up to ~98%, and the mean grain size of this layer is ~2.1 μm. Both the ~1.05 mm depth layers and central layers of the 1000-4 sample show partial recrystallized structures similar to the surface layer of the 850-5 sample. The grain size of the recrystallized grains in the ~1.05 mm depth layer varies from ~1.2 μm to ~2.7 μm, while most of the recrystallized grains in the central layer are smaller than ~1 μm. According to the misorientation angle map (Figure 2b), a fraction of the low angle grain boundary (*fLAGB*, 2~15°) for the 1000-4 sample increases from ~1.96% to ~27.3% as the depth increases (*fLAGB* of the ~1.05 mm depth layer is ~19.1%), along with the *fΣ3* decreases from ~53.5% to ~26.7% then to ~12.8%. Obviously, there is a significant difference in *fLAGB* and *fΣ3* in the 1000-4 sample between layers. In comparison to the 850-5 and 1000-4 samples, the difference in *fLAGB* and *fΣ3* between the surface layer and central layer diminishes remarkably as the heating temperature of the laser-irradiated surface increases to ~1050 °C, which is indicated by the KAM maps of the 1050-5 sample (Figure 1(g2)–(i2)). In particular, the *fLAGB* of the surface layer and ~1.05 mm depth layer is ~2.1% and ~3.5%, and the *fΣ3* for these two layers are ~47.3% and ~46.7%, respectively. In addition, the volume fraction of the recrystallized grains accounts for ~96.9% and ~95.3% of the surface layer and ~1.05 mm depth layer layers for the 1050-5 sample, respectively, and the content of the non-recrystallized grains further increases to ~18.3% when the depth extends to ~1.75 mm.

In general, these three samples are all characterized by decreasing grain size along the depth direction, which is accompanied by the increased volume fraction of the non-recrystallized grains and decreased content of the recrystallized grains. Furthermore, the 850-5 sample possesses a much narrower gradient layer, while the *fLAGB* and *fΣ3* are nearly the same in the surface and central layers for the 1050-5 sample.

**Figure 3:**
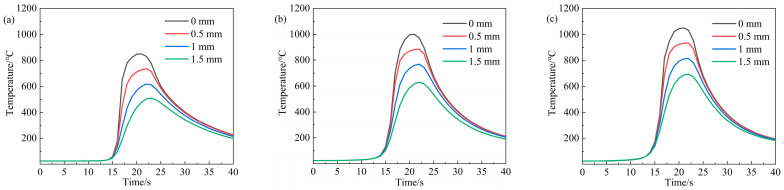
Finite element simulation of the temperature-time curves under various depths: (**a**) 850-5 sample; (**b**) 1000-4 sample [7]; (**c**) 1050-5 sample.

In order to clarify the primary reason for the diverse microstructural evolution of these cold-rolled CoCrFeMnNi HEAs, the simulated temperature fields along the depth of 850-5, 1000-4, and 1050-5 samples are shown in Figure 3. Considering the microstructural characteristics are closely related to the thermal distribution induced by laser irradiation, the formation of the inverse gradient-grained structures in these three samples can be mainly attributed to the gradient-distributed maximum heating temperature. In other words, all the simulated temperature curves exhibit a decreasing trend in peak temperatures along the depth from the top surface, which yields a significant grain growth difference in various layers, leading to the formation of the inverse gradient-grained structures. On the one hand, the maximum heating temperature of the layer with a depth of ~1 mm in the 850-5 sample is ~610 °C, whereas the peak temperature for the ~1 mm deep layer of the 1050-5 sample reaches up to ~810 °C. According to our previous research, the microstructure of the samples that annealed at ~610 °C is quite similar to that of the cold-rolled sheets, which are mainly composed of a number of lamellar deformed bands and nano/submicron-scaled grains. Simultaneously, only the recrystallized grains can be observed when annealed at ~800 °C [7]. Hence, the relatively small volume fraction of the gradient layer in the 850-5 sample is mainly caused by the low heating temperature at the ~1.05 mm depth layer, while the enhanced maximum heating temperature reduces the difference in *fLAGB* and *fΣ3* between the surface and central layers in the 1050-5 sample. On the other hand, the maximum heating temperatures are ~760 °C and ~635 °C when the depths reach ~1 mm and ~1.5 mm, causing partial recrystallization in the ~1.05 mm depth layer and central layer, which contributes to the relatively thick gradient layer in the 1000-4 sample.

### 3.2. Mechanical Property

**Figure 4:**
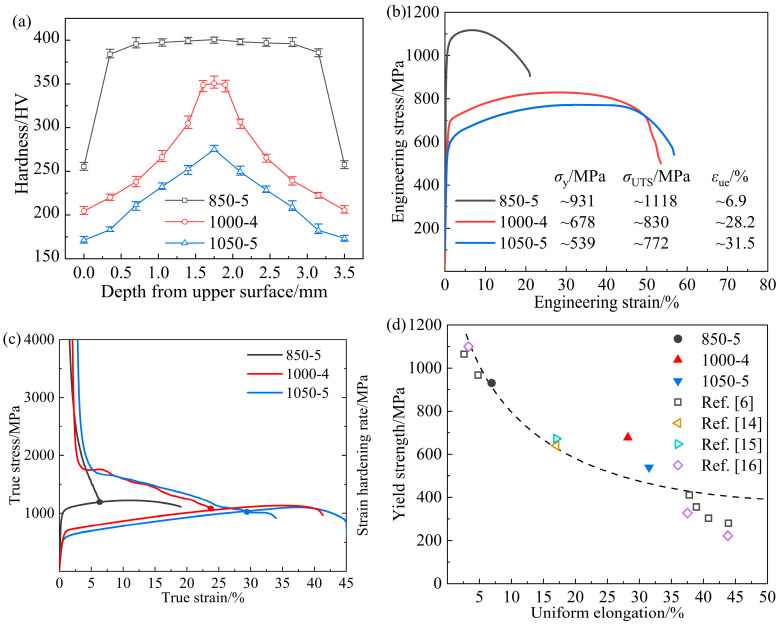
Microhardness and tensile properties of the 850-5, 1000-4, and 1050-5 samples: (**a**) distribution characteristics of the microhardness along the depths; (**b**) engineering stress–strain curves; (**c**) true stress–strain curves and corresponding strain hardening rates curves; (**d**) strength–ductility combination obtained from the present study and other references [7,15,16,17].

The cross-sectional microhardness profile of the experimental CoCrFeMnNi HEAs irradiated by various laser surface heat treatment process parameters are illustrated in Figure 4a. Gradient layers with decreasing microhardness along the depth for the 850-5, 1000-4, and 1050-5 samples are ~0.5 mm, ~1.6 mm, and ~1.75 mm, respectively. It is obvious that the distribution characteristics of the microhardness are consistent with the observed microstructures, as shown in Figure 1. For the 1000-4 sample, the microhardness difference between the top surface (~0 mm) and the central layer (~1.75 mm) reaches ~146 HV, which is close to that of the 850-5 sample (~154 HV) and significantly larger than that of the 1050-5 sample (~104 HV).

Figure 4b presents the tensile properties of the cold-rolled CoCrFeMnNi HEAs treated by various laser surface heat treatment parameters, along with the yield strength ($\sigma_{y}$), ultimate tensile strength ($\sigma_{UTS}$), and uniform elongation ($\varepsilon_{ue}$) concluded in it. The 850-5 sample shows the highest strength ($\sigma_{y}$ of ~931 MPa) but poor ductility ($\varepsilon_{ue}$ of ~6.9%). On the contrary, although the $\varepsilon_{ue}$ of the 1050-5 sample improves significantly, the superior ductility is acquired by the sacrifice of its strength. It is worth noting that the 1000-4 sample exhibits a better synergy of strength ($\sigma_{y}$ of ~678 MPa) and ductility ($\varepsilon_{ue}$ of ~28.2%). To further investigate the strain hardening behavior of these samples, the strain hardening rates of the 850-5, 1000-4, and 1050-5 samples are demonstrated in Figure 4c. Apparently, the strain hardening rate of the 850-5 sample shows a steep drop through the whole process of tensile deformation, the plastic instability at the early stage of deformation upon yielding may be attributed to its high percentage of nano/submicron-scaled grains [4]. While for the 1000-4 and 1050-5 samples, both the strain hardening curves decrease at a relatively slow rate. It is noteworthy that the strain hardening rate of the 1000-4 sample maintains a similar value to that of the 1050-5 sample at all strains, which contributes remarkably to its excellent ductility as the high strain hardening rate can prevent early necking [14].

Figure 4d summarizes the mechanical properties of the CoCrFeMnNi HEAs in this study with the inverse gradient-grained structures and other corresponding references with various microstructures. Both the 1000-4 and 1050-5 samples evade the strength–ductility trade-off curve, which is presented by the black dotted line in banana shape, suggesting the outstanding strength–ductility combinations can be acquired by the inverse gradient-grained structure, especially the 1000-4 sample. Although the 850-5 sample demonstrates the inverse gradient-grained structure as well, the strength–ductility combination of it still falls into the usual strength–ductility trade-off curve, which may be ascribed to the negligible HDI stress and hardening from the relatively narrow gradient layer. Hence, it can be speculated that increased thicknesses of gradient layers can significantly improve the synergy of strength and ductility.

### 3.3. Microstructural Evolution during Tensile Deformation

**Figure 5:**
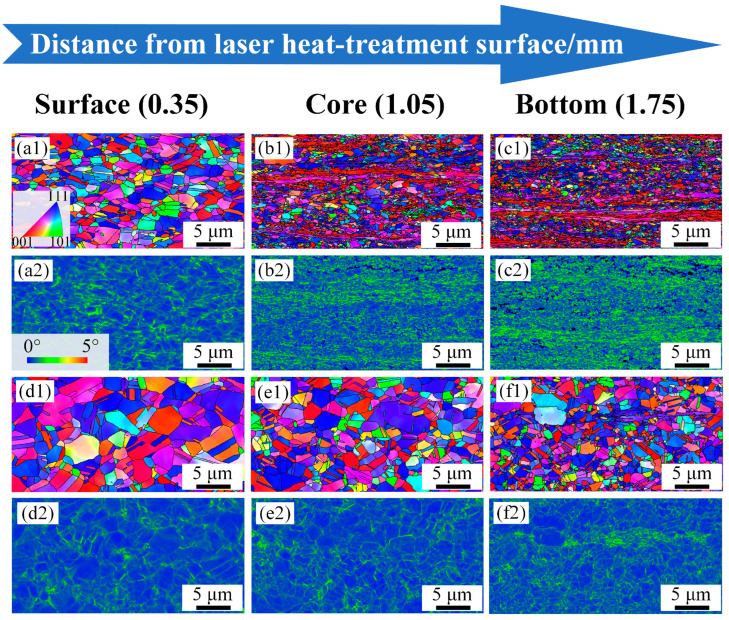
Microstructural characteristics of the inverse gradient-grained CoCrFeMnNi HEA under the tensile strain of ~15%: (**a**–**c**) IPF maps and corresponding KAM maps of the 1000-4 sample; (**d**–**f**) IPF maps and corresponding KAM maps of the 1050-5 sample.

**Figure 6:**
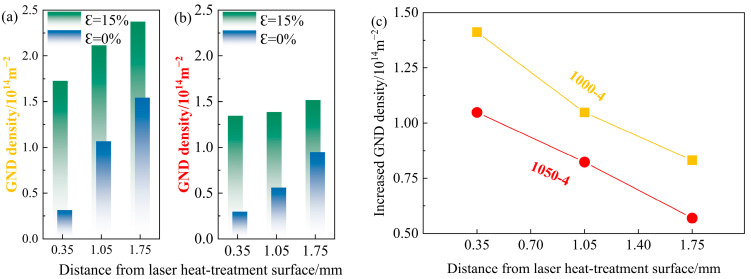
GND density evolution of the 1000-4 and 1050-5 samples during deformation estimated by the KAM values: (**a**) GND density of the 1000-4 sample at the strain of 0% and ~15% in different layers; (**b**) GND density of the 1050-5 sample at the strain of 0% and 15% in different layers; (**c**) increment of the GND density under the strain varied from 0% to ~15%.

As indicated by the hardness distribution map in Figure 4a, microhardness increases in various degrees along the depth of the 1000-4 and 1050-5 samples, leading to an entirely different mechanical incompatibility between layers. To clearly elucidate the evolution of the microstructures in different layers during deformation, the EBSD observations, including the IPF maps and corresponding KAM maps of the 1000-4 and 1050-5 samples at a strain of ~15%, are demonstrated in Figure 5. Both these deformed samples show a similar increasing trend of the KAM values along the depth to that of the undeformed samples in Figure 1. It is noteworthy that the grains of the 1000-4 sample are flat and elongated along the tensile direction (Figure 5(a1)), while those of the 1050-5 sample remain equiaxed, suggesting a more appreciable deformation occurs in the inverse gradient-grained structure with greater mechanical incompatibility between the surface and central layers, which can be identified by the evolution of geometrically necessary dislocation (GND) from the 0% strain to ~15% strain (as shown in Figure 6). Generally, the KAM value is regarded as a reflection of local misorientation [18], which can be roughly conducted for the calculation of the GND density, and the relationship between them is shown as follows [14].

$\rho_{GND}$ is the GND density, μ represents the unit length and b is the Burger’s vector (~0.255 nm for CoCrFeMnNi HEA [19]).

(1) $$\rho_{GND}=\frac{2KAM}{\mu b}$$

Apparently, there is a remarkable difference in the increment for the GND density at various layers in both the 1000-4 and 1050-5 samples, as indicated by Figure 6a,b. According to the evolution of the GND density summarized in Figure 6c, the increment of GND density in the 1000-4 sample is higher than that of the 1050-5 sample, which contributes to a more visible deformation of grains in the 1000-4 sample. Moreover, the increment of the GND density in these two samples exhibits gradient-distributed characterization along the depth. Compared to the central layer with higher hardness, the surface layer can generate more GND.

The accumulation of the GNDs In the surface layer is related to the strength difference between the surface and central layers. In particular, at the beginning stage of deformation, both the surface and central layer deform elastically without a strain gradient [20]. By increasing the strain, the surface layer will step into the yield stage earlier than the central layer due to its lower strength, leading to the formation of an elastic–plastic interface [20,21]. Along with increased strain, the elastic–plastic interface gradually migrates to the central layer [22]. In the process of the migration of the elastic–plastic interface, more GNDs will be generated and gather around this interface to maintain the continuity of the adjacent area. Hence, the increment of the GND density is higher in the surface and the ~1.05 mm depth layers. In addition, the elastic–plastic interface with a larger strength differential on both sides will generate a higher strain gradient, which needs to be accumulated by more GNDs, causing a higher increment of the GND density in the 1000-4 sample compared to the 1050-5 sample at the same strain.

**Figure 7:**
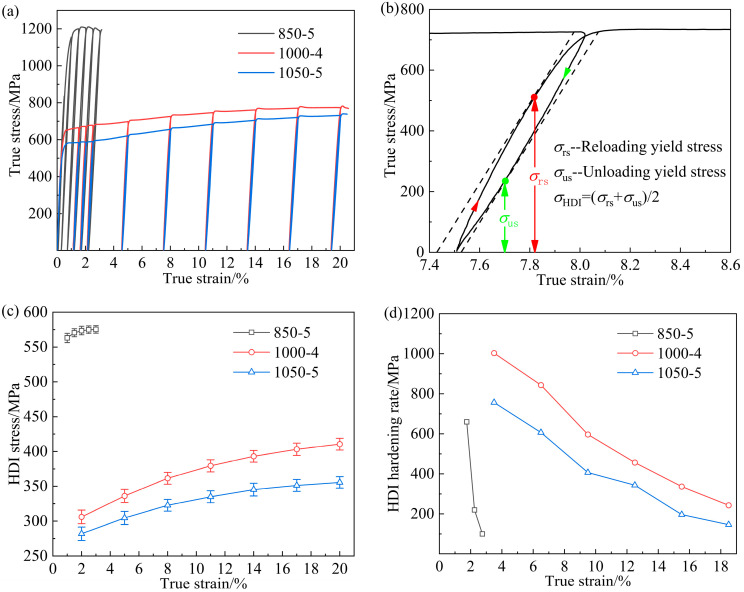
(**a**) Loading–unloading–reloading curves of the 850-5, 1000-4, and 1050-5 samples; (**b**) enlarged hysteresis loop of the 1000-4 sample; (**c**,**d**) comparison of the HDI stress and hardening rate.

Accompanied by the accumulation of the GNDs, forward stress in the relatively harder layer and back stress in the relatively softer layer are produced accordingly [17,23], which collectively generate HDI strengthening and hardening [24], leading to the good strength–ductility combination for the inverse gradient-grained CoCrFeMnNi HEA [25]. To quantitively investigate the evolution of HDI strengthening and hardening during deformation, the true stress–strain curves under the loading–unloading–reloading tensile tests of the experimental CoCrFeMnNi HEAs with inverse gradient-grained structures are presented in Figure 7a. The calculation of the HDI stress is conducted as follows [26].

$\sigma_{HDI}$ represents the HDI stress, $\sigma_{rs}$ and $\sigma_{us}$ represents reloading stress and unloading stress, respectively.

(2) $$\sigma_{HDI}=\frac{\sigma_{rs}+\sigma_{us}}{2}$$

The measuring method of $\sigma_{rs}$ and $\sigma_{us}$ is shown in Figure 7b, and the results of $\sigma_{HDI}$ for the inverse gradient-grained CoCrFeMnNi HEAs at different strains are performed in Figure 7c. The 1000-4 sample exhibits higher HDI stress than that of the 1050-5 sample during the whole process of deformation. In particular, the $\sigma_{HDI}$ of the 1000-4 and 1050-5 samples are ~396 MPa and ~345 MPa at a strain of ~15%, which is related to the higher GND density of the 1000-4 sample. Despite the 850-5 sample demonstrating the highest HDI stress and yield strength among these samples, the sharply decreased HDI hardening rate at an early stage of deformation leads to its poor ductility [27], as indicated by the HDI hardening rate curves (defined as the derivative of $\sigma_{HDI}$) in Figure 7d. On the contrary, the HDI hardening rates of the 1000-4 and 1050-5 samples are higher than ~500 MPa before the strain reaches ~8%, which contribute prominently to their enhanced mechanical properties [28,29].

**Figure 8:**
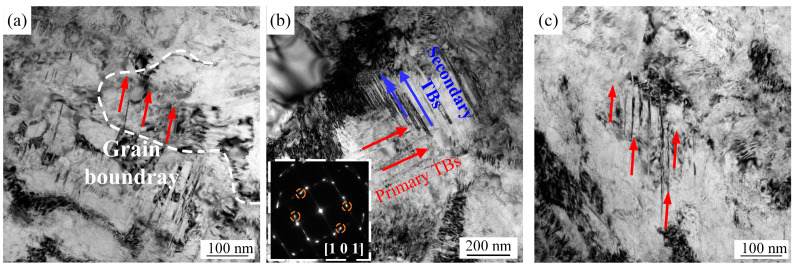
TEM observations of the core layer of the experimental CoCrFeMnNi HEAs with various inverse gradient-grained structures after tensile deformation: (**a**) 850-5 sample; (**b**) 1000-4 sample; (**c**) 1050-5 sample.

Figure 8 shows the bright field TEM images of the central layers of the 850-5, 1000-4, and 1050-5 samples after tensile deformation. A mass of nanotwins orientate consistently in the 850-5 and 1050-5 samples, whereas the two-order nanotwins are demonstrated in the 1000-4 sample. Compared to the one-order nanotwins, hierarchical nanotwins can hinder the movement of dislocations and provide more space for dislocations to accumulate, thus the hierarchical nanotwins will result in a better strength–ductility combination. Previous studies suggest that the gradient structure can effectively convert the uniaxial internal stress to multiaxial internal stress, especially for the gradient-structured material with high HDI stress [17,28]. Usually, this multiaxial stress state facilitates more slip systems for dislocations and promote the formation of hierarchical nanotwins [30]. However, hierarchical nanotwins are rarely observed in the 850-5 and 1050-5 samples, even these two samples have the inverse gradient-grained structure. In other words, not all gradient structures can prompt the generation of hierarchical nanotwins. For the 850-5 sample, most of the observed one-order nanotwins in its central layer derive from the previous process of cold-rolling. This is because the non-recrystallized grains of its central layer require extremely high critical stress to activate twinning [31]. For the 1050-5 sample, the process of laser surface heat treatment induces the detwinning of its original deformed microstructure [32], which results in few nanotwins in the central layer before loading. Without the initial one-order nanotwins, the nucleation of the subordinate nanotwins can hardly take place [33]. Hence, the partial recrystallized structure with a great deal of one-order nanotwins is of benefit to the formation of hierarchical nanotwins. That is, the reserved initial nanotwins and the grain size of the partial recrystallized structure for the 1000-4 creates favorable conditions for the formation of hierarchical nanotwins.

The relatively superior strength–ductility combination, which is obtained by the inverse gradient-grained structure as the 1000-4 sample, may benefit from the following significant factors. First of all, the central layer owns the partial recrystallized microstructure with low-level recrystallization, which contributes to the high mechanical incompatibility between the central layer and the coarse-grained zone at the surface layer. Generally, the magnitude of the strain gradient increases with larger mechanical incompatibility [9], and the GND density is proportional to the strain gradient [10]. Considering that the HDI strengthening and hardening are mainly caused by the GND pile-up [12], it can be easily deduced that the relatively hard microstructure in the central layer with a large strength difference compared with the surface layer may enhance the strength–ductility combination. Furthermore, this partial recrystallized microstructure in the central layer induces hierarchical nanotwins, which also improves the strength–ductility synergy. Finally, the gradient layer should occupy a large volume fraction due to the enhanced effect of the strength and ductility being limited as the thickness of layer with structural gradient decreases. According to the above-mentioned design principles of the inverse gradient-grained structure, repeated scanning accompanied by the appropriate laser power and scanning speed may further optimize the strength–ductility combination. This is mainly because the grain coarsening occurs gradually at the surface layer without sacrificing the microhardness of the central layer along with the increased number of laser irradiation [7]. In order to verify this idea and promote the laser surface heat treatment process as a promising method to fabricate the gradient-structured materials, the technological parameters of laser surface heat treatment will be adjusted in our future work, especially the scanning times.

## 4. Conclusions

In this work, the microstructural characteristics and tensile properties of the experimental CoCrFeMnNi HEAs with various inverse gradient-grained structures were investigated in the laboratory. Based on the detailed discussion of strength–ductility mechanisms, some design principles of the inverse gradient-grained structure are proposed.

Three samples with different gradient-distributed microstructural characteristics were obtained by laser irradiation under different parameters. The relatively low heating temperature leads to a much narrower gradient layer in the 850-5 sample, whereas the excessive heating temperature reduces the difference in *fLAGB* and *fΣ3* between the hard core and soft surface in the 1050-5 sample. Significantly, a relatively thick gradient layer with large mechanical incompatibility is obtained in the 1000-4 sample.

Apart from the 850-5 and 1050-5 samples, the 1000-4 sample evades the strength–ductility trade-off. The limited volume fraction of the gradient-grained layer accompanied by a large amount of deformed microstructure at the central layer results in the poor ductility of the 850-5 sample. The strength–ductility combination of the 1000-4 sample is better than that of the 1050-5 sample, which mainly resulted from the abundant GNDs nucleated from the interface between the hard central layer and soft surface layer as well as the hierarchical nanotwins generated at the core layer.

According to the characteristics of the structural gradients for the 1000-4 sample, maintaining high microhardness with a low-degree recrystallization in the central layer, facilitating the hierarchical nanotwins in the core layer, and enlarging the volume proportion of gradient layer are the key ways to improve the strength–ductility combination of the inverse gradient-grained CoCrFeMnNi HEAs.

## Data Availability

Data are contained within this article.
